# The impact of HIV knowledge and attitudes on HIV testing acceptance among patients in an emergency department in the Eastern Cape, South Africa

**DOI:** 10.1186/s12889-020-09170-x

**Published:** 2020-07-06

**Authors:** Sofia Ryan, Elizabeth Hahn, Aditi Rao, George Mwinnyaa, John Black, Roshen Maharaj, Nomzamo Mvandaba, Yandisa Nyanisa, Thomas C. Quinn, Bhakti Hansoti

**Affiliations:** 1 The Johns Hopkins University, Baltimore, MD USA; 2 The Johns Hopkins Bloomberg School of Public Health, Baltimore, MD USA; 3 Division of Intramural Research, National Institute of Allergy and Infectious Diseases, Baltimore, MD USA; 4 Department of Infectious Disease, Livingstone Hospital, Port Elizabeth, South Africa; 5 Department of Medicine, Faculty of Health Sciences, Walter Sisulu University, Mthatha, South Africa; 6 Department of Emergency Medicine, Livingstone Hospital, Port Elizabeth, South Africa

**Keywords:** HIV testing, HIV attitudes, HIV knowledge, Emergency departments, Testing acceptance

## Abstract

**Background:**

Transmission of HIV in South Africa continues to be high due to a large proportion of individuals living with undiagnosed HIV. Uptake of HIV testing is influenced by a multitude of factors including the patient’s knowledge and beliefs about HIV.

**Methods:**

This study sought to quantify the impact of knowledge and attitudes on HIV testing acceptance in an emergency department by co-administering a validated HIV knowledge and attitudes survey to patients who were subsequently offered HIV testing.

**Results:**

During the study period 223 patients were interviewed and offered HIV testing. Individuals reporting more negative overall attitudes (*p* = 0.006), higher levels of stigma to HIV testing (*p* < 0.001), and individuals who believed their test was confidential (*p* < 0.001) were more likely to accept an HIV test.

**Conclusions:**

Interventions focused on improving patient perceptions around testing confidentiality will likely have the greatest impact on testing acceptance in the emergency department.

## Background

People living with undiagnosed HIV are major contributors to the continued transmission of the virus [1]. Consequently, accessing HIV testing remains a critical step in meeting the UNAIDS 90–90-90 targets [2, 3]. South Africa has long advocated for universal testing and since 2010 has a national Provider-Initiated Counseling and Testing (PICT) policy in place, which promotes the routine availability of free HIV testing across all healthcare facilities [4]. In recent years, the percentage of South Africans who know their HIV status has increased from 50.0% in 2008 to 66.5% in 2015 [4]. Furthermore, in 1997 South Africa introduced the Life Orientation (LO) curriculum in secondary schools to raise awareness about preventing sexually transmitted infections (STIs) and the need for HIV testing [5]. However, testing uptake, especially in missed populations such as young men, remains low, wherein less than 70% of South Africans aware of HIV counseling and testing services have accessed these services [4, 6]. There appears to be a discrepancy between where HIV testing resources are being channeled and where they are most needed.

Multiple studies have demonstrated the impact of HIV knowledge and attitudes towards HIV testing in South Africa, where stigma and low rates of risk perception were noted as significant barriers to HIV testing [7–12]. Likewise, individuals with more negative attitudes towards HIV were less likely to seek testing [10]. Other studies in sub-Saharan Africa have shown similar findings relating low perceived risk of infection, fear of test results, and stigma to refusal of an HIV test [13–15]. In addition to the impact of attitudes on HIV testing, inadequate access to education serves as a barrier to the acceptance of an HIV test [6]. However, a study in KwaZulu-Natal found that the LO curriculum resulted in a significant increase in HIV knowledge among learners [16].

Emergency Departments (EDs) serve populations with a high burden of undiagnosed HIV in both the United States [17, 18] and in developing countries [19–21]. The Eastern Cape province is responsible for 16% of South Africa’s new HIV infections and high prevalence rates of HIV are present in EDs [22, 23]. Unfortunately, HIV testing is not routinely implemented in EDs, likely due to a lack of trained staff and inadequate resources [24, 25]. This study aims to explore the impact of HIV knowledge and attitudes on HIV testing acceptance among ED patients in the Eastern Cape province of South Africa. The findings from this study will help inform the routine implementation of HIV testing in ED settings.

## Methods

### Study design

This study combines survey responses regarding HIV knowledge and attitudes with patient data on HIV testing acceptance among patients in an ED. The study was conducted at Livingstone Hospital in the Eastern Cape of South Africa from June 4th to July 15th, 2018 as part of the larger Walter Sisulu Infectious Diseases Screening in the Emergency Department (WISE) study. The aim of the WISE study is to implement HIV testing as per the national guidelines in the Eastern Cape and to quantify the burden of HIV among patients in this setting [23]. Primary data collection of the HIV Knowledge and Attitudes Survey (HKAS) was conducted concurrently with the WISE study during a three-week period between June 18th and July 8th, 2018.

### Setting

Most hospitals and clinics in the Eastern Cape are overcrowded, understaffed, lacking resources, and poorly managed [26]. Livingstone Hospital is a provincial tertiary hospital situated in the Korsten suburb of Port Elizabeth, South Africa and forms part of the Port Elizabeth Hospital Complex. The hospital provides 24-h emergency care, including trauma services, to the Port Elizabeth area and receives referrals from regional and district hospitals and clinics from its catchment area. Currently, there are no dedicated HIV counselors present in the ED at Livingstone Hospital, requiring medical officers and nurses to take on this responsibility. The ED has 50 beds and 15 doctors managing an average annual volume of 32,000 patients. The hospital serves both walk-in patients and patients arriving by ambulance.

### Life orientation curriculum

The LO curriculum was introduced into the South African national curriculum in 1997 in an effort to help learners develop life skills and make responsible decisions about their health. The LO curriculum is initiated from grade 4 in the Foundation phase and is compulsory for South African students in grades 10 through 12. Part of the curriculum aims to educate learners about sexually transmitted infections, risky sexual behaviors, and HIV prevention. Two hours per week are dedicated to the curriculum, totaling 80 h of instruction in each grade level in which the curriculum is implemented [5].

### Recruitment

Patients were recruited by HIV Counseling and Testing (HCT) staff from the waiting room of the emergency department and were verbally asked if they would spend 10–15 min completing a brief questionnaire about their thoughts around HIV testing. All adult patients aged 18 years or older who were clinically stable and agreed to participate in the study were eligible for enrollment. Patients were excluded from the study if they were minors, unable to give informed consent due to decreased levels of consciousness or critically ill status, or patients returning to the ED who had been enrolled previously. Critically ill patients were defined as those with a South African Triage Scale (SATS) score of ‘emergency’ [27]. HCT staff only approached patients who were initially assigned an ‘emergency’ SATS score after their condition was stabilized in the ED.

### Data collection

Data collection occurred through two parallel processes. Information on patient knowledge and attitudes towards HIV, demographics, and exposure to the LO curriculum was collected through the HKAS conducted via convenience sampling of patients enrolled in the WISE study. HCT staff aimed to survey five to ten patients per day. HCT staff briefly introduced the survey and obtained verbal consent before proceeding. The questions and answer options were dictated to the patient by HCT staff in English, Afrikaans, or Xhosa, and responses were recorded on electronic tablets. To capture HIV testing data, HCT staff approached eligible patients presenting to the ED once the triage process was completed, so as not to interfere with patient care. Patients were informed of the ongoing study and offered a rapid, point-of-care (POC) HIV test. Written informed consent was sought for HCT. Data on age, sex, SATS score, chief complaint, past medical history, clinical course, and HIV status, were recorded using case report forms. HCT staff also noted if the patient accepted or refused an HIV test, the test results, and the patient’s reasoning for accepting or refusing the test.

### Survey instrument

**Fig. 1:**
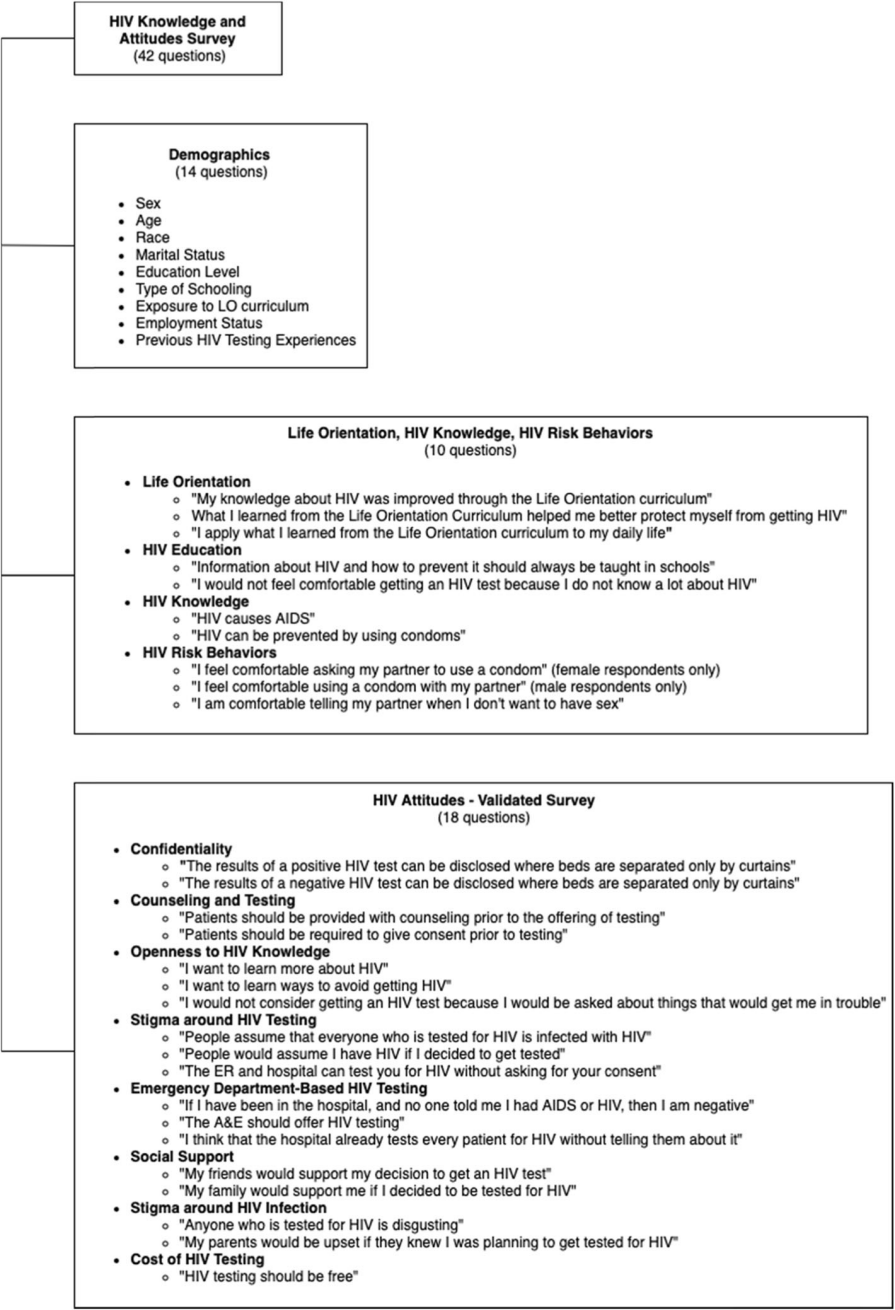
HIV Knowledge and Attitudes Survey

The HKAS consisted of forty-two questions assessing patient demographics, education level, exposure to the LO curriculum, and HIV knowledge and attitudes. The HKAS was created on the Qualtrics© survey platform (Qualtrics, Provo, UT). Questions about patient demographics, LO exposure, HIV knowledge, and POC test status were developed specifically for this study. The 18 attitudes questions in the HKAS were derived from a previously validated survey instrument and were published in the HKAS with permission from the rights holder [28]. A pooling of 43 HIV attitudes questions were tested in ED populations to develop a validated survey for this context. Surveys were conducted among English or Xhosa-speaking patients in South African EDs, and answers were recorded in English. Exploratory factor analysis was used to determine correlation patterns between individual questions and Cronbach alpha scores were calculated. The fewest number of questions that represented the maximum variation from the original pool were chosen, resulting in an 18-question validated survey. Cognitive assessments were not conducted during the validation process. The HIV attitudes survey instrument has previously been used to assess attitudes among English-speaking patients in an ED in East London, in the Eastern Cape of South Africa [7]. A breakdown of the survey questions used in this study can be found in Fig. 1. The full HKAS has been included in the supplementary material as Additional File 1. Knowledge and attitudes questions on the survey were recorded using a 5-point Likert scale with 1 being ‘strongly disagree’ and 5 being ‘strongly agree.’ Negatively worded questions were reversed in numeric value, so the number 5 consistently reflected positive attitudes. Two knowledge questions, “HIV causes AIDS” and “HIV can be prevented by using condoms,” were included in the analysis for this study. Responses to the knowledge questions were categorized as ‘correct’ or ‘incorrect’, wherein ‘strongly agree’ or ‘agree’ were grouped as ‘correct’ and ‘no opinion’, ‘disagree’, or ‘strongly disagree’ were grouped as ‘incorrect.’

### Data analysis

The primary outcome measure of this study was the effect of HIV knowledge and attitudes on testing acceptance. Case report forms were scanned and uploaded onto DataFax© (DataFax, Clinical DataFax Systems Inc., Hamilton, Ontario, Canada) to facilitate data validation. HKAS data on Qualtrics were imported into Stata v.15 (StataCorp LLC, Texas) for analysis. Patient survey responses were linked to their corresponding case report form using a unique study identification number. This facilitated the linking of patient acceptance or refusal of an HIV test to their responses on the surveys for further analysis. Patients reporting a known HIV positive status were removed from analysis.

Scored responses for the eighteen attitudes questions were summed to create an overall attitude score, and separate scores were calculated for each of the thematic attitude groups listed in Fig. 1. A ‘perfect’ overall attitudes score was defined as a score of 90 (scoring a 5 on every question), while an ‘overall positive’ attitudes score was defined as a score of 72 or higher (scoring an average of 4 or above on every question). HKAS scores were analyzed as binary variables, where a score of 72 for overall attitudes was considered to be a ‘positive’ attitude towards HIV and a score less than 72 was considered to be a ‘negative’ attitude towards HIV. For the thematic attitudes groups the cut-off score for the binary variable was 8, with the exception of four categories; the cut-off score for Openness to HIV knowledge, HIV testing stigma, and ED-based HIV was 12, and the cut-off score for Cost of HIV Testing was 4. The descriptive titles of the binary variables for the thematic attitude groups, represented in Table 2, are based on the content of the questions within the specific sections of the survey.

Analysis was conducted using chi-square tests to explore individual associations between HIV knowledge indicators, attitudes scores, and testing acceptance. Simple logistic regressions and two multiple logistic regression models were used to examine the crude and adjusted odds of accepting a POC HIV test. The first multiple logistic regression was adjusted for age and gender, while the second multiple logistic regression was adjusted for age, gender, race, and attitude score in each of the seven HKAS attitude domains. One participant was excluded from the attitudes analysis because they refrained from answering all eighteen attitudes questions on the HKAS. However, the patient answered the two knowledge questions and was therefore included in the analysis of the two knowledge questions. A total of 26 (11.66%) survey participants did not answer at least one survey question. In a sensitivity analysis, imputation was used for missing attitude scores for each category and the overall score, and our findings did not change. Due to the convenience sampling approach of the HKAS, an a priori sample size could not be determined.

## Results

Over the four-week study period, WISE study staff approached 873 patients, of which 819 (93.8%) agreed to participate in the study. Of the 819 patients enrolled in the WISE study, 91 (11.1%) patients reported a known diagnosis of HIV and were removed from analysis. A complete demographic profile of the remaining 728 patients enrolled in the WISE study is presented in Table 1, stratified by completion of the HKAS and acceptance of a POC test. Of the 728 patients enrolled in the WISE study, 223 (30.6%) completed the HKAS and 505 (69.4%) did not. A higher proportion of patients accepted a HIV test in the group that completed the HKAS (77.1%) as compared to the group that did not complete the HKAS (68.7%). No significant differences were found between those who completed and did not complete the HKAS and those who accepted and declined a POC test with regard to sex and presenting complaint. Patients who accepted a POC test were more likely to have an ‘urgent’ or ‘routine’ SATS score compared to a ‘very urgent’ or ‘emergency’ score among both those who completed and did not complete the HKAS survey, and this difference was statistically significant (*p* = 0.021). A higher proportion of patients over the age of 40 years declined the HKAS survey but were more likely to accept a POC test in both the completed HKAS and declined HKAS groups (*p* = 0.040). Among those who completed the HKAS survey, a higher proportion of coloured (26/123, 21.1%) and white (6/19, 31.6%) patients declined testing compared to their black (15/76, 19.7%) counterparts (*p* = 0.03). Exposure to the Life Orientation curriculum among patients who completed the HKAS was not shown to have a significant impact on testing behaviors (*p* = 0.575) compared to testing behaviors in those without LO exposure.

**Table 1:** Demographic profile of study participants stratified by HKAS completion and POC test acceptance

	Completed HKAS		Declined HKAS		Total (***n*** = 728)	Chi-squared (***p***-value)
	Accepted POC test (***n*** = 172)	Declined POC test (***n*** = 51)	Accepted POC test (***n*** = 347)	Declined POC test (***n*** = 158)		
**Sex**						
Male	101 (58.7%)	28 (54.9%)	196 (56.5%)	73 (46.2%)	398 (54.7%)	6.172 (0.104)
Female	71 (41.3%)	23 (45.1%)	151 (43.5%)	85 (53.8%)	330 (45.3%)	
**Age**						
< 20 Years	12 (7.0%)	0 (0.0%)	14 (4.0%)	7 (4.4%)	33 (4.5%)	17.59 (0.040)*
20–29 Years	52 (30.2%)	18 (35.3%)	103 (29.7%)	32 (20.3%)	205 (28.2%)	
30–39 Years	36 (20.9%)	6 (11.8%)	90 (25.9%)	44 (27.8%)	176 (24.2%)	
≥ 40 Years	72 (41.9%)	27 (52.9%)	140 (40.4%)	75 (47.5%)	314 (43.1%)	
**South African Triage Score (SATS)**						
Emergency	0 (0.0%)	1 (2.0%)	4 (1.1%)	5 (3.2%)	10 (1.4%)	19.60 (0.021)*
Very Urgent	8 (4.7%)	5 (9.8%)	25 (7.2%)	5 (3.2%)	43 (5.9%)	
Urgent	102 (59.3%)	35 (68.6%)	180 (51.9%)	83 (52.5%)	400 (54.9%)	
Routine	62 (36.0%)	10 (19.6%)	138 (39.8%)	65 (41.1%)	275 (37.8%)	
**Chief Complaint**						
Medical	84 (48.8%)	27 (52.9%)	160 (46.1%)	66 (41.8%)	337 (46.3%)	2.658 (0.447)
Trauma	88 (51.2%)	24 (47.1%)	187 (53.9%)	92 (58.2%)	391 (53.7%)	

* Designates significance at the *p* < 0.05 level

There was a significant difference in testing acceptance based upon overall attitudes score and the categories of confidentiality, HIV testing stigma, and social support when stratified by HIV testing acceptance status, as shown in Table 2. Additionally, individuals with higher levels of stigma around HIV testing were also more likely to accept a POC test (*p* < 0.001). Individuals who believed their test was confidential and who had higher levels of social support were more likely to accept a POC test (*p* < 0.001 and *p* = 0.018, respectively).

**Table 2:** HKAS score as a binary variable, stratified by POC test acceptance

	Accepted POC Test (***n*** = 172)	Declined POC Test (***n*** = 51)	Total (***n*** = 223)	Chi Squared (***p***-value)
**Overall Attitudes Score (%)**				
Negative	117 (82.98%)	24 (17.02%)	141	7.4360 (0.006)*
Positive	55 (67.07%)	27 (32.93%)	82	
**Categorical Attitude Scores (%)**				
**Confidentiality**				
Do not believe test is confidential	69 (66.35%)	35 (33.65%)	104	12.8485 (< 0.001)**
Believe test is confidential	103 (86.55%)	16 (13.45%)	119	
**Counseling & Testing**				
Disapprove of counseling before HIV testing	24 (75.00%)	8 (25.00%)	32	0.0961 (0.757)
Approve of counseling before HIV testing	148 (77.49%)	43 (22.51%)	191	
**Openness to HIV Knowledge**				
Do not want to learn more about HIV	48 (77.42%)	14 (22.58%)	62	0.0041 (0.949)
Want to learn more about HIV	124 (77.02%)	37 (22.87%)	161	
**HIV Testing Stigma**				
High stigma around HIV testing	103 (87.29%)	15 (12.71%)	118	14.6600 (< 0.001)**
Low stigma around HIV testing	69 (65.71%)	36 (34.29%)	105	
**ED-based HIV Testing**				
Disapprove of ED-based HIV testing	123 (74.55%)	42 (25.45%)	165	2.4025 (0.121)
Approve of ED-based HIV testing	49 (84.48%)	9 (15.52%)	58	
**Social Support**				
Low levels of social support	16 (59.26%)	11 (40.74%)	27	5.5617 (0.018)*
High levels of social support	156 (79.59%)	40 (20.41%)	196	
**HIV Infection Stigma**				
High stigma around HIV infection	37 (77.08%)	11 (22.92%)	48	0.0001 (0.993)
Low stigma around HIV infection	135 (77.14%)	40 (22.86%)	175	
**Cost of HIV Testing**				
Disapprove of free HIV testing	2 (66.67%)	1 (33.33%)	3	0.1887 (0.664)
Approve of free HIV testing	170 (77.27%)	50 (22.73%)	220	

* Designates significance at the < 0.05 level

** Designates significance at the < 0.001 level

The odds of testing acceptance among individual attitudes domains are presented in Table 3. SATS was found to significantly affects odds of testing acceptance (OR 0.459, *p* = 0.007) and was therefore included in the adjusted models. Patients who believed their test was confidential were on average three times more likely to accept a test compared to patients who did not believe their test was confidential (in unadjusted models OR = 3.26, *P* < 0.001, when adjusting for age and sex OR = 3.27, *p* = 0.001, and when adjusting for age, sex, race, SATS, and the other attitudes categories OR = 2.57, *p* = 0.016). Participants with higher levels of social support also had significantly higher odds of testing acceptance (in unadjusted models OR = 2.68, *P* = 0.022 and models adjusting for age, sex, and SATS OR = 2.88, *p* = 0.018). Participants with higher levels of social support also had higher odds of testing acceptance in models adjusting for age, sex, SATS, race, and other attitudes categories (OR = 2.38), but the difference was not significant (*p* = 0.076). However, notably participants with lower levels of stigma around HIV testing were less likely to accept a test than participants with higher levels of stigma around HIV testing, and this finding was significant in unadjusted models (OR = 0.279, *p* < 0.001), models adjusting for age, sex, and SATS (OR = 0.285, *p* < 0.001), and models adjusting for age, sex, SATS, race, and the other attitudes categories (OR = 0.247, *p* = 0.002). Most other positive attitudes tested in the model were associated with higher odds of testing acceptance but were not significant in unadjusted or adjusted models.

**Table 3:** Odds of HIV test acceptance by attitude category

	Unadj. OR	95% CI	***p***-value	Adj. OR^a^	95% CI	***p***-value	Adj. OR^**b**^	95% CI	***p***-value
Confidentiality (Believe test is confidential)	3.26	1.68–6.35	< 0.001^‡^	3.01	1.52–5.93	0.001^†^	2.48	1.15–5.37	0.021^†^
Counseling & Testing (Approve of counseling before HIV testing)	1.14	0.481–2.74	0.757	1.17	0.478–2.84	0.736	2.08	0.718–6.01	0.177
Openness to HIV knowledge (Want to learn more about HIV)	0.977	0.486–1.97	0.949	0.939	0.454–1.94	0.865	1.47	0.600–3.61	0.398
Stigma around HIV testing (Low levels of stigma around HIV testing)	0.279	0.142–0.548	< 0.001^‡^	0.285	0.143–0.566	< 0.001^‡^	0.247	0.103–0.600	0.002^†^
ED based HIV testing (Approve of ED-based testing)	1.86	0.842–4.11	0.125	1.61	0.711–3.64	0.253	0.975	0.388–2.45	0.957
Social support (High levels of social support)	2.68	1.15–6.23	0.022^†^	2.88	1.20–6.95	0.018^†^	2.38	0.914–6.20	0.076
Stigma around HIV infection (Low stigma around HIV infection)	1.00	0.469–2.14	0.993	0.923	0.421–2.02	0.842	1.08	0.426–2.74	0.868
Cost of HIV testing (Approve of free HIV testing)	1.70	0.151–19.1	0.668	1.39	0.106–18.0	0.803	1.08	0.065–17.8	0.959

^a^Adjusted for age, sex, and SATS

^b^Adjusted for age, sex, SATS, race, and other attitude categories

† Statistically significant result at the < 0.05 level

‡ Statistically significant result at the < 0.001 level

The majority of participants correctly answered the knowledge question “HIV can be prevented by using condoms” (210/223, 94.2%). A significantly higher proportion (*p* < 0.001) of HKAS participants who accepted a POC test correctly answered the knowledge question “HIV causes AIDS” (126/172, 73.3%) compared to those who declined a POC test (23/51, 45.1%). Overall, individuals with higher levels of HIV knowledge were more likely to accept an HIV test, especially those who knew that HIV can lead to AIDS.

## Discussion

The purpose of our study was to determine the impact of HIV knowledge and attitudes on HIV testing acceptance in a busy ED setting in Port Elizabeth, South Africa. Our study found testing acceptance to be influenced by individual attitudes in regard to confidentiality concerns, social support, and HIV testing stigma. Confidentiality concerns and HIV testing stigma significantly predict the odds of testing acceptance, even when controlling for all other attitude domains. However, patient demographics, their level of education, and exposure to the LO curriculum were not found to be significant predictors of testing acceptance.

Knowledge of HIV in our study population was good, overall the majority of patients in our study knew HIV could be prevented by condoms and that HIV causes AIDS. We did find that a large portion of our sample were exposed to the LO curriculum (35.9%) (although this did not produce significant differences in testing acceptance). While the LO teaching in school may have contributed to knowledge, it may not directly impact testing acceptance due to the time gap between when participants were exposed to LO and when they were enrolled in our study. Furthermore, health promotion messaging around HIV testing is extremely prominent in South Africa, so isolating the impact of a single educational intervention is challenging. Lastly, there are numerous documented challenges of implementing the LO curriculum, including lack of formal teacher training, traditional views of sexuality among teachers, and large class sizes [29].

Our study identified that patients who believed testing was confidential were on average three times more likely to accept testing compared to those who did not and determined beliefs around confidentiality to be the most significant modifiable attitude to improve HIV testing uptake. Participants in our study may have been more likely to accept an HIV test despite high levels of stigma due to greater trust of ED providers. Hansoti et al. found that 61.5% of patients in an ED in the Eastern Cape agreed that they trusted HIV testing counselors to keep their information private and confidential [7]. This finding suggests that patients may feel more comfortable accepting a test in an ED setting, despite displaying higher levels of stigma towards HIV testing, since they believe that the HIV testing counselor will protect information about their HIV status. Additionally, other studies in sub-Saharan Africa and Europe have shown fear of a breach in confidentiality to be a major barrier to HIV testing, especially among the youth [7, 30–32]. Furthermore, implementation of confidential testing practices (such as separate rooms) were recommended to increase testing acceptance based on a study offering PICT in public community health centers in South Africa [33]. A strategy to increase testing acceptance may be to address confidentiality concerns upfront by assuring patients that their test is completely confidential and that their results will not be disclosed to others. Promoting public awareness and education on the importance of confidential HIV testing and non-disclosure of results to others may also be a worthwhile strategy to increase testing acceptance.

Individuals in our study were also more likely to accept a POC test if they felt their peers and family would be supportive of their choice to receive an HIV test. This finding was not significant when controlling for other attitudes factors, suggesting that the interaction of social support with other attitudes domains may affect testing acceptance rather than social support alone. However, this finding remains important to consider since it was one of the few attitudes categories that remained significant in unadjusted models and models adjusting for age and sex. A systematic review of 42 papers from 13 countries in sub-Saharan Africa demonstrated that the support of social networks was an important factor in the decision to take an HIV test and that fear of losing social support after a positive result was a barrier to HIV testing [12]. A study of loss to care after HIV diagnosis in South Africa found that patients referred by healthcare providers for an HIV test were more likely to be lost to care after a positive diagnosis, likely because were less prepared to deal with the consequences of their HIV-positive status [34]. The authors suggested that physicians should combine HIV test referrals with the necessary social support strategies to improve retention in care after a positive diagnosis [34]. Healthcare providers can further address the role of social support in future HIV testing interventions by advocating for post-test support groups for HIV positive patients and their families or peers.

Unexpectedly, participants in this study with higher levels of stigma around HIV testing (i.e., more likely to assume that everyone who is tested for HIV has HIV or more likely to believe people would assume they have HIV if they are seen being tested) had higher odds of accepting an HIV test than those with lower levels of stigma. This is contrary to other studies in sub-Saharan Africa, which have reported that higher rates of stigma around HIV testing are associated with lower testing acceptance [9, 14]. Our questions focused on stigma around HIV testing as opposed to stigma towards people living with HIV. Stigma was assessed in our study based on constructs relating to people’s perceptions of those who test for HIV (e.g. “People assume that everyone who is tested for HIV has HIV”) and beliefs about whether the EDs had the ability to test people for HIV without their knowledge. In other studies, stigma was defined either through people’s perceptions of people living with HIV (e.g. “people with HIV/AIDS are dirty”) and the violence they might face (e.g. “people with HIV/AIDS face verbal abuse”) [9] or more broadly as negative attitudes towards people living with HIV [14]. Perhaps the wording in our study tapped into the privacy concerns around HIV testing and the ability to test in a place where it was universally offered and where other individuals from the patients’ communities were unlikely to be present. These differences in how stigma was defined may explain the differences in levels of stigma between people who accepted an HIV test in our study versus in others.

There is also a long-standing history of mistrust of government-run testing programs due to years of apartheid rule and a history of AIDS denialism [35]. A study by Bogart et al. assessed testing behavior among patients at STD clinics and suggested that conspiracy beliefs that the government is giving people HIV through testing programs have decreased support and participation in government-sponsored testing programs [36]. Likewise, government-run AIDS awareness programs have historically lacked credibility which have affected South African’s interactions with them [37]. It is possible that participants in our study may have been more likely to accept an HIV test despite higher levels of HIV testing stigma due to a combination of greater trust of ED providers, as mentioned previously, and the provision of testing in the context of a non-governmental research program.

Surprisingly, this study also found that overall attitudes scores were higher among HKAS participants who did not accept an HIV test (72.23 vs 70.01, *p* = 0.006). This is contrary to the current literature, which states that more negative attitudes are associated with refusal of an HIV test or never having had an HIV test in sub-Saharan Africa [10, 38], and that accepting an HIV test is associated with more positive attitudes towards testing [39] and less stigmatizing attitudes towards people living with HIV [40]. This may be due to the fact that the attitudes around stigma, confidentiality and social support more heavily influenced patients’ decision to test in our study, and that the overall attitude score does not account for the weight of these individual attitudes in their decision making. This is demonstrated in the multi-logistic regression model in Table 3, where both adjusted and unadjusted odd ratios show an independence of these individual domains on testing acceptance.

Several of the attitudes in our analysis (such as counseling and testing, openness to HIV knowledge, ED-based HIV testing, stigma around HIV infection, and cost of HIV testing) did not significantly impact testing acceptance, nor were the odds of testing acceptance significant when these categories were considered independently of one another. However, this does not mean that these factors are not important considerations when planning to implement a testing program, they remain a fundamental part of the HIV testing process and likely play a role in the complex decision-making process around testing acceptance. It is also possible that concerns such as cost and counseling may have been addressed by HCT staff during the HIV testing intervention, given that we implemented the South African national HIV testing guidelines. However, the constructs of confidentiality and social support were shown to be modifiable barriers to testing, and if addressed appropriately could improve testing uptake in this setting.

One limitation of this study is that less than a third (27.7%) of patients approached by HCT counselors during the study period completed the HKAS. This is likely due to the time-consuming nature of the survey and the rapid flow of patients through the ED. HKAS participants may be more likely to be easily accessible by HCT counselors, in less pain, and able to spend extra time completing the questions with the counselor. Patients may have refused to take the HKAS for similar reasons to refusing an HIV test in the parent WISE study, including pain and time constraints. However, the current study would have been strengthened if reasons for not completing the HKAS has been recorded. The strengths of this study include its potential for informing future HIV testing policy and improving the practice of HIV testing in South African health facilities. Likewise, this study samples a unique population of individuals who have a higher burden of HIV, likely because they do not seek out testing. This is extremely beneficial for designing future HIV testing interventions to encourage greater amounts of individuals to test.

## Conclusion

To our knowledge, this is the first study to use a validated instrument to examine the effects of demographics, the LO curriculum, knowledge, and attitudes on HIV testing acceptance in an emergency department setting in South Africa. Studies in South Africa have shown favorable patient responses to ED-based HIV testing [7] and the PICT process has been successful in normalizing the HIV testing process and increasing uptake [11, 12]. Though testing practices have improved, there is still high variability in testing acceptance by demographic characteristics and education levels. Our study shows that interventions focused on improving testing confidentiality and social support should be the basis of future testing programs.

## Supplementary information

**Additional file 1.** Full HIV Knowledge and Attitudes Survey. Description of data: The file contains all questions used in the HKAS during data collection. The file includes question and answers options as well as display and skip logic for relevant questions.

## Abbreviations

ED: Emergency Department
HCT: HIV Counseling and Testing
HKAS: HIV Knowledge and Attitudes Survey
LO: Life Orientation
PICT: Provider-Initiated Counseling and Testing
POC: Point-of-Care
SATS: South African Triage Scale
STI: Sexually Transmitted Infection
WISE: Walter Sisulu Infectious Disease Screening in the Emergency Department
