# Supplementary material for: The impact of HIV knowledge and attitudes on HIV testing acceptance among patients in an emergency department in the Eastern Cape, South Africa
Source: BMC Public Health. 2020 Jul 6;20:1066. doi: 10.1186/s12889-020-09170-x (PMC7339484; doi:10.1186/s12889-020-09170-x)
Supplement: Supplementary file 1 [file 12889_2020_9170_MOESM1_ESM.docx]

HIV South Africa- Patient Attitudes and Knowledge

Start of Block: Demographics

Q28 Study ID number

________________________________________________________________

Q1 Are you:

- Male  (1) 

- Female  (2) 

- Other  (3) 

Q2 Which of these Race Categories fit you? (you may check more than one if you identify with more than one race group)

- Black African  (1) 

- White  (2) 

- Coloured  (3) 

- Indian/Asian  (4) 

- Other  (5) ________________________________________________

Q3 What is your age?

- < 20 years old  (1) 

- 20-29 years old  (2) 

- 30-39  years old  (3) 

- ≥ 40 years old  (4) 

Q4 What is your marital status?

- Married  (1) 

- Widowed  (2) 

- Divorced  (3) 

- Separated  (4) 

- Single  (5) 

Q5 What is your education level?

- Less than secondary school education  (1) 

- Completed some or all of secondary school  (2) 

- Matriculated from secondary school  (3) 

- University degree or above  (4) 

Q6 What kind of school did you attend?

- Private  (1) 

- Public  (2) 

- Government  (3) 

- No schooling  (4) 

Display This Question:

If What is your education level? != Less than secondary school education

Q7 Have you been exposed to the Life Orientation curriculum during your secondary schooling?

- Yes  (1) 

- No  (2) 

- Unsure  (3) 

Display This Question:

If Have you been exposed to the Life Orientation curriculum during your secondary schooling? = Yes

Q20 Do you remember learning about HIV in secondary school through the Life Orientation curriculum?

- Yes  (1) 

- No  (3) 

- Unsure  (4) 

Q8 Are you employed?

- Yes  (1) 

- No  (2) 

Q10 Have you ever had an HIV test at a clinic?

- Yes  (1) 

- No  (2) 

Q11 Have you ever been tested for HIV somewhere other a clinic?

- No  (1) 

- Yes  (2) ________________________________________________

Q12 How long ago was your last HIV test?

- Within the last 2 months  (1) 

- Between 2 and 6 months ago  (2) 

- Between 6 and 12 months ago  (3) 

- More than 12 months ago  (4) 

- I don't know  (5) 

- I've never been tested for HIV  (6) 

Display This Question:

If How long ago was your last HIV test? != I've never been tested for HIV

Q14 What was the result of your last HIV test?

- Positive  (1) 

- Negative  (2) 

- I don't know  (3) 

Display This Question:

If What was the result of your last HIV test? = Positive

Q17 Are you:

- Currently on ART  (1) 

- Not taking ART  (2) 

Q27 For each statement below, please tell us your opinion: 

|  | Strongly Agree (1) | Agree (2) | Neither Agree nor Disagree (3) | Disagree (4) | Strongly disagree (5) |
| --- | --- | --- | --- | --- | --- |
| I would not feel comfortable getting an HIV test because I do not know a lot about HIV (1) |  |  |  |  |  |
| Information about HIV and how to prevent it should always be taught in schools (2) |  |  |  |  |  |
| I am comfortable telling my partner when I don't want to have sex (3) |  |  |  |  |  |
| Have you been exposed to the Life Orientation curriculum during your secondary schooling? = Yes What I learned through the Life Orientation curriculum helped me better protect myself from getting HIV (4) |  |  |  |  |  |
| Are you: = Female I feel comfortable asking my partner to use a condom (5) |  |  |  |  |  |
| Have you been exposed to the Life Orientation curriculum during your secondary schooling? = Yes I apply what I learned in the Life Orientation curriculum to my daily life (6) |  |  |  |  |  |
| Have you been exposed to the Life Orientation curriculum during your secondary schooling? = Yes My knowledge about HIV was improved through the Life Orientation curriculum (7) |  |  |  |  |  |
| HIV can be prevented by using condoms (8) |  |  |  |  |  |
| HIV causes AIDS (9) |  |  |  |  |  |
| Are you: = Male I feel comfortable using a condom with my partner (10) |  |  |  |  |  |

End of Block: Demographics

Start of Block: Knowledge and Attitudes

Q22 For each statement below, tell us your opinion:

|  | Strongly Agree (1) | Agree (2) | Neither agree nor disagree (3) | Disagree (4) | Strongly disagree (5) |
| --- | --- | --- | --- | --- | --- |
| I want to learn more about HIV (1) |  |  |  |  |  |
| The results of a positive HIV test can be disclosed when beds are separated only by curtains (2) |  |  |  |  |  |
| I would not consider getting an HIV test because I would be asked about things I have done that could get me in trouble (3) |  |  |  |  |  |
| People assume that everyone who is tested for HIV is infected with HIV (4) |  |  |  |  |  |
| I think that the hospital already tests every patient for HIV without telling them about it (5) |  |  |  |  |  |
| My friends would support my decision to get an HIV test (6) |  |  |  |  |  |
| The results of a negative HIV test can be disclosed when beds are separated only by curtains. (7) |  |  |  |  |  |
| HIV testing should be free (8) |  |  |  |  |  |
| My family would support me if I decided to be tested for HIV (9) |  |  |  |  |  |
| Anyone who is tested for HIV is disgusting (10) |  |  |  |  |  |
| Patients should be provided with counseling prior to the offering of testing (11) |  |  |  |  |  |
| Patients should be required to give consent prior to testing. (12) |  |  |  |  |  |
| I want to learn ways to avoid getting HIV. (13) |  |  |  |  |  |
| People would assume I have HIV if I decided to get tested. (14) |  |  |  |  |  |
| My parents would be upset if they knew I was planning to get tested for HIV (15) |  |  |  |  |  |
| The emergency department should offer HIV testing (16) |  |  |  |  |  |
| If I have been in the hospital, and no one told me that I have AIDS or HIV, then I am negative (17) |  |  |  |  |  |
| The ER and hospital can test you for HIV without asking for your consent (18) |  |  |  |  |  |

Q33 HIV testing status

- Accepted Test  (1) 

- Declined Test  (2) 

Display This Question:

If HIV testing status = Accepted Test

Q35 HIV test result

- Positive  (1) 

- Negative  (2) 

- Indeterminate  (3) 

Q34 HIV status (from CRF)

- Known positive  (1) 

- New positive  (2) 

- Negative  (3) 

- Unknown  (4) 

Display This Question:

If HIV testing status = Declined Test

Q36 Why did patient refuse to take an HIV test 

- Patient does not trust test  (1) 

- Tme constraint  (2) 

- Previous negative test experience  (3) 

- Does not believe they are at risk for HIV  (4) 

- Already aware of HIV status  (5) 

- Patient tested within the last year  (6) 

- Patient does not want to know  (7) 

- Patient does not want others to know they are being tested for HIV  (8) 

- Other  (9) ________________________________________________

End of Block: Knowledge and Attitudes
